# Frequent visitors in a university psychiatric emergency department in Greece

**DOI:** 10.1192/j.eurpsy.2022.775

**Published:** 2022-09-01

**Authors:** I. Vlachos, P. Chondraki, P. Magioglou, E. Lempesi, D. Bourazana, C. Papageorgiou, M. Margariti

**Affiliations:** 1 National and Kapodistrian University of Athens Medical School, Eginition Hospital, First Department Of Psychiatry, Athens, Greece; 2 National and Kapodistrian School of MedicineUniversity of Athens, First Department Of Psychiatry “eginition” Hospital, Athens, Greece

**Keywords:** substance use disorder, frequent visitors, psychosocial problems, Psychiatric emergencies

## Abstract

**Introduction:**

Background :The profile of “frequent visitors” at the psychiatric emergencies (PE) has not been sufficiently investigated in Greece.

**Objectives:**

In this study we aimed to investigate the prevalence and relevant parameters of frequent PE visits in a Greek University Psychiatric Hospital for the year 2017.

**Methods:**

In a retrospective study, we analyzed data of patients who presented in the PE of Eginition University Hospital in Athens during 2017. Frequent visitors were grouped under this category if they had at least five visits per year. Clinical and sociodemographic data of the patients were further related to number of visits.

**Results:**

84 patients were characterized as frequent visitors carrying out 9.8% of the total number of visits. 50% were women and 70% of them were living with family members. Anxiety, depressive and psychotic symptoms were the most frequent major complaints at the time of their visit, whereas psychosocial problems were associated with increased number of visits. Moreover, in terms of the underlying diagnosis substance use disorders significantly related to more frequent visits

**Conclusions:**

Psychosocial problems and the diagnosis of substance use disorders significantly correlated to the number of visits at the PE of a university hospital setting in Greece for 2017.

**Disclosure:**

No significant relationships.

